# Photocorrosion of WO_3_ Photoanodes in Different Electrolytes

**DOI:** 10.1021/acsphyschemau.1c00004

**Published:** 2021-05-19

**Authors:** Julius Knöppel, Attila Kormányos, Britta Mayerhöfer, André Hofer, Markus Bierling, Julien Bachmann, Simon Thiele, Serhiy Cherevko

**Affiliations:** † Forschungszentrum Jülich GmbH, Helmholtz Institute Erlangen-Nürnberg for Renewable Energy (IEK-11), Forschungszentrum Jülich, Egerlandstr. 3, 91058 Erlangen, Germany; ‡ Department of Chemical and Biological Engineering, Friedrich-Alexander-Universität Erlangen-Nürnberg, Egerlandstr. 3, 91058 Erlangen, Germany; § Department of Chemistry and Pharmacy, Chemistry of Thin Film Materials, IZNF, Friedrich-Alexander-Universität Erlangen-Nürnberg, Cauerstr. 3, 91058 Erlangen, Germany; ∥ Institute of Chemistry, Saint Petersburg State University, 198504 Saint Petersburg, Russian Federation

**Keywords:** Photocorrosion, Tungsten oxide, Photostability, Water splitting, Electrode kinetics

## Abstract

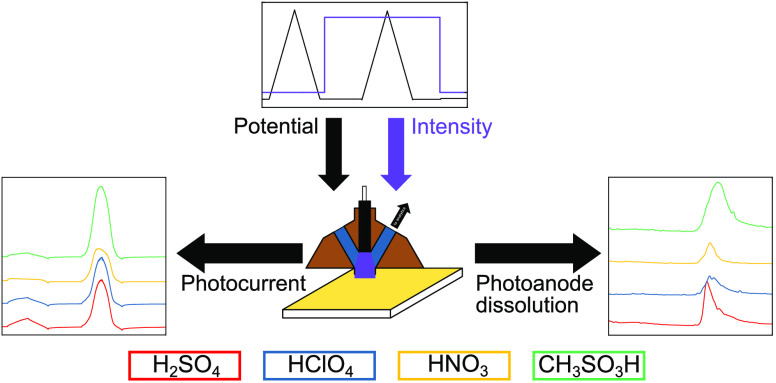

Photocorrosion of an n-type semiconductor is anticipated to be unfavorable if its decomposition potential is situated below its valence band-edge position. Tungsten trioxide (WO_3_) is generally considered as a stable photoanode for different photoelectrochemical (PEC) applications. Such oversimplified considerations ignore reactions with electrolytes added to the solvent. Moreover, kinetic effects are neglected. The fallacy of such approaches has been demonstrated in our previous study dealing with WO_3_ instability in H_2_SO_4_. In this work, in order to understand parameters influencing WO_3_ photocorrosion and to identify more suitable reaction environments, H_2_SO_4_, HClO_4_, HNO_3_, CH_3_O_3_SH, as electrolytes are considered. Model WO_3_ thin films are fabricated with a spray-coating process. Photoactivity of the samples is determined with a photoelectrochemical scanning flow cell. Photostability is measured in real time by coupling an inductively coupled plasma mass spectrometer to the scanning flow cell to determine the photoanode dissolution products. It is found that the photoactivity of the WO_3_ films increases as HNO_3_ < HClO_4_ ≈ H_2_SO_4_ < CH_3_O_3_SH, whereas the photostability exhibits the opposite trend. The differences observed in photocorrosion are explained considering stability of the electrolytes toward decomposition. This work demonstrates that electrolytes and their reactive intermediates clearly influence the photostability of photoelectrodes. Thus, the careful selection of the photoelectrode/electrolyte combination is of crucial importance in the design of a stable photoelectrochemical water-splitting device.

## Introduction

In light of the current climate crisis, our dependency on the efficient utilization of renewable energy becomes inevitable. Solar energy is the renewable energy source with the highest potential for harvesting. In fact, an area of 2% of the world’s landmass covered with solar modules would be sufficient to meet the whole population’s energy requirements. However, the intermittency of solar energy increases the need for expanded energy conversion, storage, and subsequent utilization, e.g., in transportation and chemical industries. As promising approaches toward energy storage, generation of hydrogen and other value-added products via photoelectrochemical (PEC) water splitting and CO_2_ reduction are commonly discussed. In PEC devices, semiconducting photoelectrodes are used to drive the relevant hydrogen evolution reaction (HER), oxygen evolution reaction (OER), and CO_2_ reduction reaction (CO2RR) directly using sunlight as a primary energy source.^1−4^ They are usually formed from inexpensive transition metals or their oxides,^5,6^ opening up possibilities for widespread application of the PEC technologies. Considering PEC water splitting, promising solar-to-hydrogen efficiencies of up to 30% have been reported so far.^7^ Hence, it is unsurprising that research interest in photoelectrochemistry is growing steadily.^8−10^ Commercialization, on the other hand, has progressed only slowly. In addition to other reasons, limited stability of PEC devices is considered as a major obstacle toward their commercialization.^11^ Indeed, while long-term durability on a scale of several thousands to tens of thousands hours is required, stable performance of modern PEC devices has only being demonstrated over several hours to days.^12−14^

Thermodynamically, the stability of metal-oxide-based semiconductors can be predicted by estimating their band gap position versus redox potentials for OER and HER or CO2RR.^5^ Thus, an n-type semiconductor is thermodynamically stable if its decomposition potential lies deep in the valence band. The other extreme case is when the decomposition potential of the semiconductor is located at a less positive potential than the water oxidation potential; in this case, the decomposition process is more favorable than the OER. Finally, there are cases when the decomposition potential of the semiconductor lies between the valence band position and the water oxidation potential. WO_3_ belongs to this category.^5^ If only thermodynamics is considered, WO_3_ is stable. Hence, WO_3_ has been a candidate for photoanodes since the beginning of the PEC water splitting research.^15^ It offers a band gap of 2.6 to 2.7 eV, which is in the visible spectrum and a suitable band position with the conduction band slightly above 0 V_RHE_.^16−18^ Furthermore, it can be easily synthesized from peroxotungstic acid precursors by electrodeposition,^19,20^ dip coating,^21^ spin coating,^22^ and inkjet printing of sol–gel-derived WO_3_ inks.^23^

The thermodynamic view on stability alone is, however, insufficient. In cases where the decomposition potential lies higher than the valence band position, a kinetic competition results between OER and photoelectrode corrosion.^5,24,25^ Furthermore, the decomposition potential of photoelectrodes should depend on electrolytes, especially if there are reactions between the electrode and products of electrolyte decomposition. Thus, high rates of WO_3_ photocorrosion during OER was recently demonstrated using a photoelectrochemical scanning flow cell coupled to an inductively coupled plasma mass spectrometer (PEC-ICP-MS).^26^ In addition to WO_3_, PEC-ICP-MS was also used to study photocorrosion of BiVO_4_^27,28^ and ZnO single crystals.^29^ In PEC-ICP-MS, an electrolyte is pumped through a PEC cell downstream to ICP-MS for elemental analysis of dissolution products. It is possible to analyze dissolution products in real time with and without light illumination. Considering WO_3_ as a model system, it was found that in an H_2_SO_4_ electrolyte dissolution is only observed under illumination.

In the current work, we aim at extending our original study by investigating the influence of different electrolytes on the operational stability of WO_3_ photoanodes. With the PEC-ICP-MS system, we determine the dissolution stability of WO_3_ photoanodes under operation in different electrolytes that are widely used in the community. The results are discussed in light of the reported behavior of WO_3_ photoanodes in these electrolytes, such as photoactivity and selectivity. It is shown that, in addition to photoelectrode materials, electrode/electrolyte combinations have to be studied to construct a stable photoelectrochemical device.

## Results and Discussion

### Synthesis and Characterization of WO_3_ Films

**Scheme 1:**
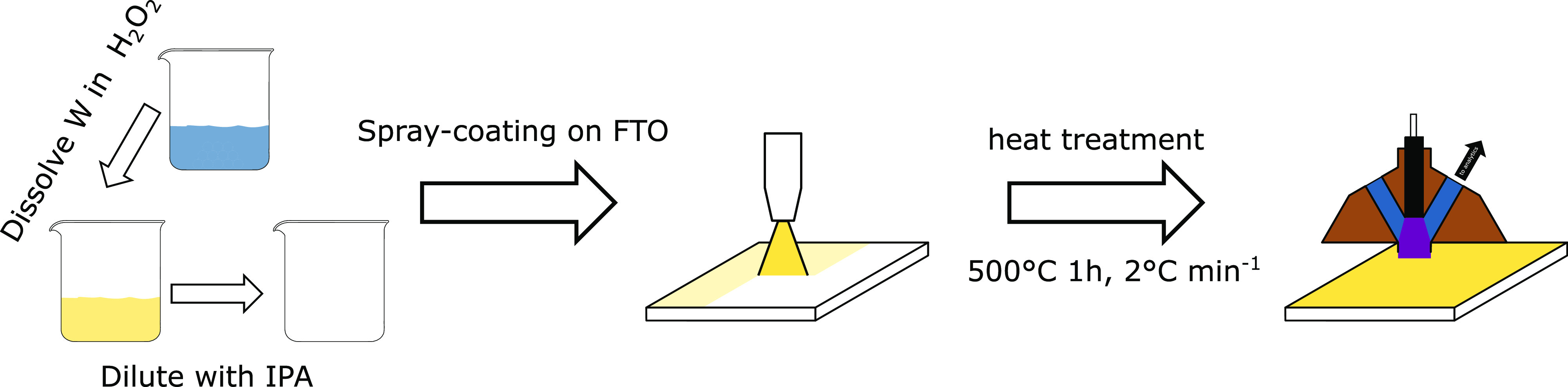
Synthesis Scheme for the WO_3_ Films Used in This Study W powder is dissolved in H_2_O_2_ and diluted with IPA. The precursor is spray-coated onto FTO-coated glass slides. After calcination, the formed WO_3_ films are measured by a photoelectrochemical scanning flow cell (PEC-SFC). A more detailed description of the PEC-SFC system can be found in the literature.^26^

A spray-coating approach was used to fabricate WO_3_ thin films based on the well-established peroxotungstic acid routes.^19,23,30^ The synthesis process is sketched in Scheme 1. Peroxotungstic acid was formed by dissolving tungsten powder in H_2_O_2_. After full dissolution and removing the excess H_2_O_2_ by heating the solution, the precursor was diluted with isopropyl alcohol (IPA) to be used as ink in the spray-coating process. For electrode preparation, the ink was spray-coated onto fluorine-doped tin oxide (FTO)-coated glass slides and exposed to a heat treatment at 500 °C for 1 h. The resulting photoelectrode area was 6.25 cm^2^, but the synthesis method allows for the preparation of thin-film photoelectrodes with an arbitrary size.

**Figure 1:**
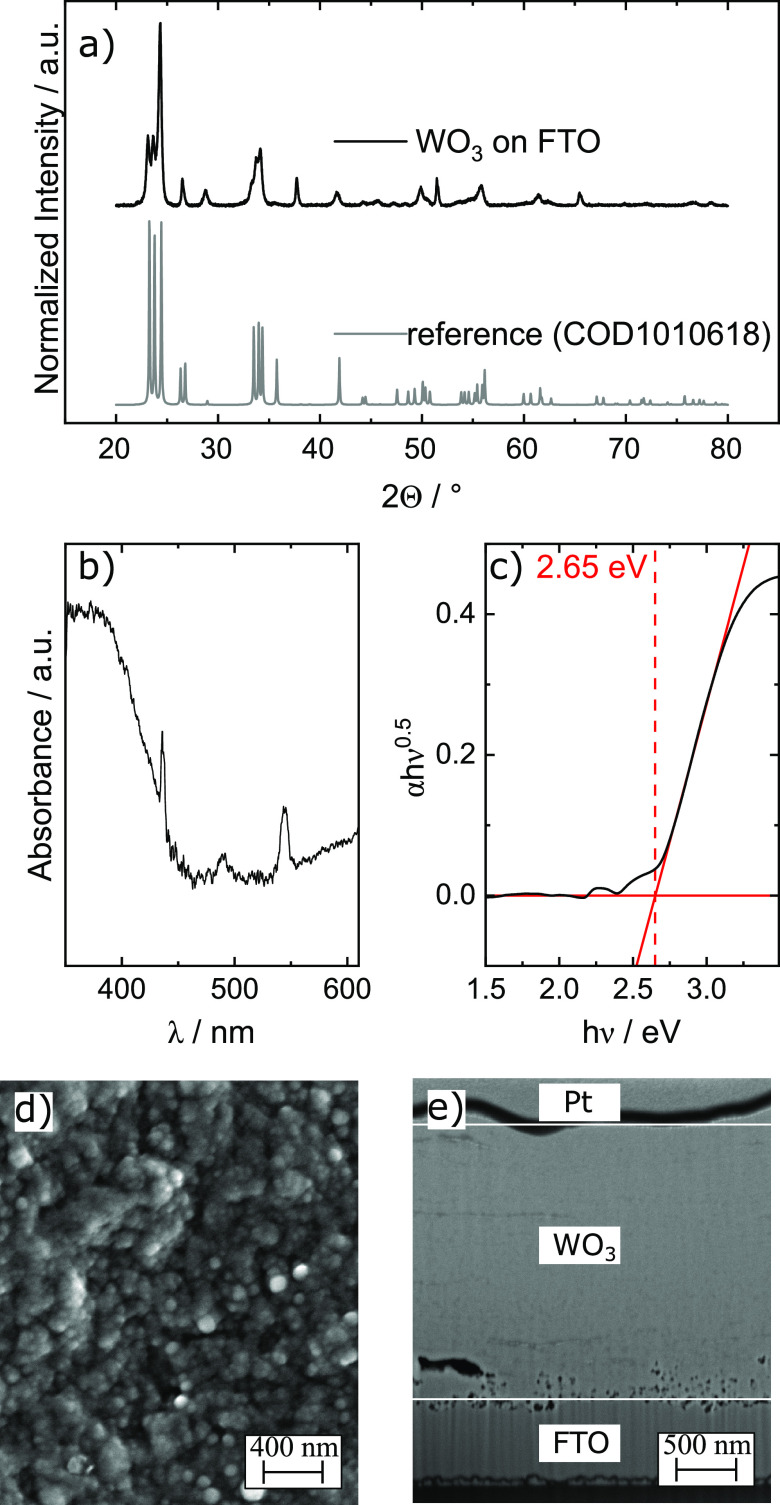
Characterization of WO_3_ films by (a) XRD spectrum of the synthesized samples. The reflectance is similar to the reference spectrum. (b) Absorption spectrum of WO_3_ thin film. (c) Baseline-corrected Tauc plot analysis. The data show an indirect transition with a band gap of 2.65 eV. (d) SEM micrograph of WO_3_ thin film. (e) SEM micrograph of a WO_3_ thin film liftout. The Pt layer was applied to protect the film during the lifout. The distance between the white lines is ∼2.3 μm.

After calcination, the films appear to have a uniform yellow surface. X-ray diffraction (XRD) analysis, shown in Figure 1a, reveals a crystalline structure. The reflections match literature data for WO_3_, published in the crystallography open database.^31^ Optoelectronic properties of the as-synthesized photoelectrodes were studied with UV–vis spectroscopy. The spectrum is shown in Figure 1b. To estimate the band gap, a Tauc analysis was performed on UV–vis data, shown in Figure 1c. It displays an indirect transition with a band gap of 2.65 eV, which is in good agreement with literature data for WO_3_.^32−35^

The morphology of the WO_3_ thin films was characterized by scanning electron microscopy (SEM) and is shown in Figure 1d. It is visible that WO_3_ exhibits a porous structure with an average pore size of around 100 nm. SEM liftouts, as shown in Figure 1e, show a film thickness of 2–3 μm. WO_3_ photoanodes in the literature show a wide range of structural features. WO_3_ electrodes synthesized via a microwave-assisted sol–gel route were around 3 μm thick with a higher porosity, similar to electrodes synthesized from commercial nanoparticles.^36,37^ On the other hand, electrodes derived from an aqueous sol–gel method show a more compact structure with an electrode thickness of around 2.5 μm.^38^ Hence, thickness and porosity are, with the spray-coating parameters used in this study, in the same ballpark as previously synthesized photoelectrodes.

**Figure 2:**
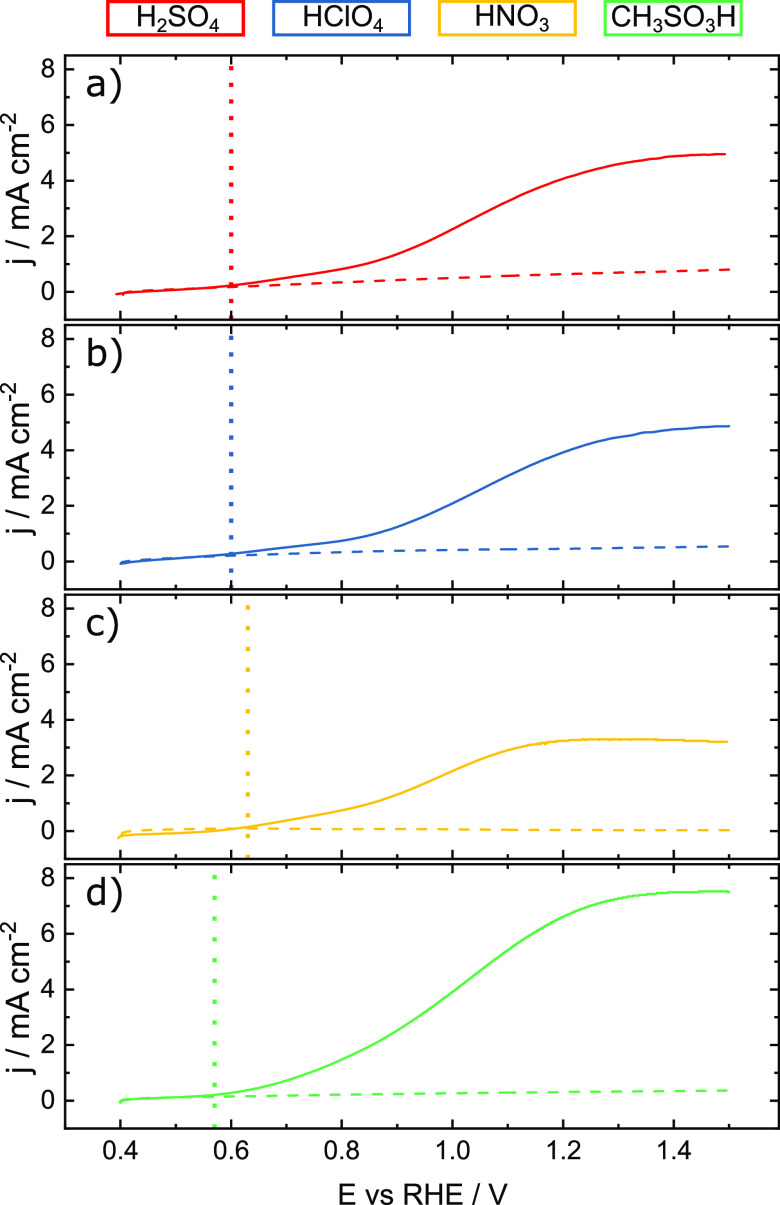
Photoelectrochemical behavior of WO_3_ films. Dark (dashed) and illuminated (solid, 50 mW cm^–2^ at λ = 385 nm) ramps were recorded with a scan rate of 10 mV s^–1^ in (a) sulfuric acid, (b) perchloric acid, (c) nitric acid, and (d) methanesulfonic acid electrolytes. The dashed, vertical lines indicate the photocurrent onset potentials.

### Photoelectrochemical Activity of WO_3_ Films in Different Electrolytes

The activity of WO_3_ films was measured in four different electrolytes with 0.1 M concentration, namely, sulfuric acid, perchloric acid, nitric acid, and methanesulfonic acid. Linear sweep voltammograms from 0.4 V_RHE_ to 1.5 V_RHE_ with a scan rate of 10 mV s^–1^ were recorded in the dark and under constant illumination with a UV light-emitting diode (LED) (385 nm, 50 mW cm^–2^). The results are presented in Figure 2.

Dark currents are negligible in all electrolytes. Under illumination, the WO_3_ films show limiting photocurrents ranging from 3 mA cm^–2^ (nitric acid) to 8 mA cm^–2^ (methanesulfonic acid). These photocurrents exceed those shown for WO_3_ in the literature by a factor of around 2.5.^37−41^ This discrepancy to literature values can be explained by the different light sources. In the literature, the typical light source is an AM1.5G solar simulator at 1 sun (100 mW cm^–2^) illumination intensity. In this work, a UV light source (λ_mean_ = 385 nm) with an intensity of 50 mW cm^–2^ was used. As WO_3_ absorbs preferentially photons in the UV spectrum, such as the employed light source, the lower monochromatic intensity used in this study leads to higher limiting photocurrents. However, the limiting photocurrents for the different electrolytes show trends similar to those in the literature.

The onset potentials vary slightly between electrolytes in the order of E^on^(CH_3_SO_3_H) < E^on^(H_2_SO_4_) ≈ E^on^(HClO_4_) < E^on^(HNO_3_). The shift in onset potential is consistent with previous reports.^39^ Onset potentials and maximum photocurrent values in different electrolytes indicate that water oxidation is not the kinetically limiting charge transfer process occurring at the surface of WO_3_. Indeed, it has been shown previously that various electrolyte anions, which are considered to be inert, can decompose due to the transfer of photogenerated holes from WO_3_.^39,40^ For example, in sulfuric acid, the dominating reaction at WO_3_ photoanodes is not OER but is the decomposition of sulfates to persulfates:^40,42−45^
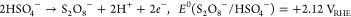
Moreover, there have been reports stating S_2_O_8_^–^ is the targeted product instead of O_2_.^39,40,42^ The pH of the electrolyte solution plays a critical role; if the pH is higher than 1, OER gradually becomes kinetically favored over the SO_4_^2–^ decomposition reaction.^45^ In methanesulfonic acid, no oxygen is produced at the photoanode.^37^ The real value for the redox couple of methanesulfonic acid, *E*^0^((CH_3_SO_3_)_2_/CH_3_SO_3_^−^), has not been reported yet but is estimated to be lower than the decomposition potential of the S_2_O_8_^–^/HSO_4_^−^ couple in the literature.^39^ In contrast to the sulfur-containing electrolytes, the decomposition potential of HClO_4_ lies at a much higher value.^40^
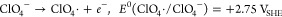
While degradation of ClO_4_^–^ is thermodynamically possible at WO_3_ electrodes, the main reaction product measured in reactors is oxygen, although enhanced H_2_O_2_ formation rates have been reported.^38,40^ In contrast to the sulfur-containing electrolytes, perchlorate radicals are not stable in an aqueous environment. It has been demonstrated previously that perchlorate radicals bind only weakly to the active sites and thus detach easily from the WO_3_ surface.^40^ In conclusion, OER is catalyzed by ClO_4_· radicals with H_2_O_2_ as an intermediate in a homogeneous manner. Therefore, close to 100% faradaic efficiency toward OER was obtained for WO_3_ in HClO_4_ solution.^40^ There have been no reports about faradaic efficiencies and dominant reactions in nitric acid. However, as N in HNO_3_ is already in its highest oxidation state, further oxidation can be excluded. We speculate that an oxidation, similar to perchloric acid with *E*^0^(NO_3_·/NO_3_^−^) similar to *E*^0^(ClO_4_·/ClO_4_^−^), takes place. Alternatively, OER might directly proceed on the electrode without an intermediate reaction, as the lowered current suggests.

As W^*n*+^ has a strong complexing affinity and as the reaction products are highly reactive peroxides, different kinetics in the presence of different investigated electrolyte anions might affect electrode stability, as well.^5,46^ In the following, the photoelectrochemical scanning flow cell (PEC-SFC) outlet was coupled to the inlet of an ICP-MS. This in situ technique allowed us to monitor the degradation of WO_3_ while performing various photoelectrochemical protocols.

### In Situ Measurements of WO_3_ Photoelectrode Stability

**Figure 3:**
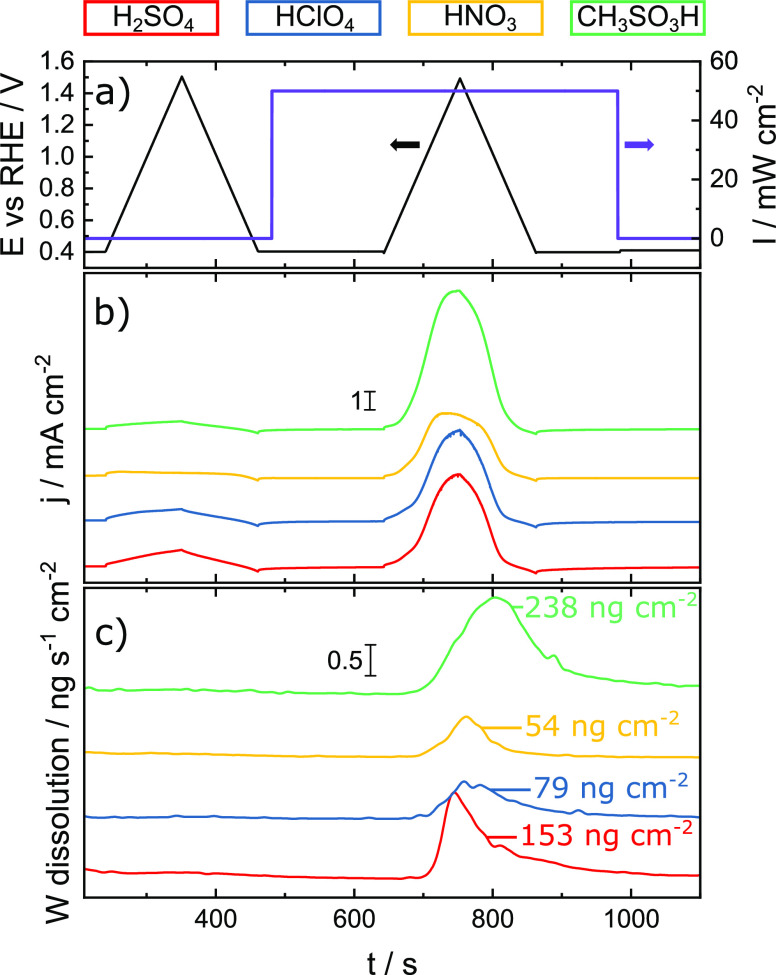
Stability measurement of WO_3_ thin film measured in different electrolytes. (a) Applied potential against the reversible hydrogen electrode and illumination intensity of monochromatic light at λ = 385 nm. (b) Resulting current. (c) Resulting W dissolution.

To study the stability of WO_3_ thin films in different electrolytes, the PEC-ICP-MS setup was engaged.^26^ Dark and illuminated (385 nm, 50 mW cm^–2^) cyclic voltammograms (CVs) at 10 mV s^–1^ were performed consecutively within the same protocol. The data for electrolytes H_2_SO_4_, HClO_4_, HNO_3_, and CH_4_O_3_S are shown in Figure 3. Dark CVs show little to no dissolution. On the other hand, the maximal dissolution rates during illuminated CVs range from 0.5 to 1.5 ng s^–1^ cm^–2^. A compact WO_3_ electrode of 2 μm thickness would, at this rate, fully decompose in several hours. The total dissolved W amount for each electrolyte is displayed in Figure 3c alongside the dissolution data. It shows that WO_3_ photocorrodes most in electrolytes, in which the highest photocurrents are measured.

The fact that no W dissolution occurs in the dark suggests that the decomposition of W should be associated with processes triggered by illumination. This is in line with previous reports where it was demonstrated that anions, considered inert before, can decompose or form reactive intermediates due to the transfer of photogenerated holes from the valence band of a semiconductor.^39,40^ Dissolution in electrolytes that form stable intermediates (sulfuric acid and methanesulfonic acid) is strongly enhanced compared to that with perchloric acid and nitric acid, where no such behavior is known. While W dissolves in an amount of 250 and 150 ng cm^–2^ during one CV in methanesulfonic acid and sulfuric acid, the total dissolved amount in perchloric acid and nitric acid is significantly below 100 ng cm^–2^. Furthermore, it appears that the shapes of the dissolution peaks vary between electrolytes. A steep incline, peaking before the peak of the photocurrent function, is observed in sulfuric acid. In perchloric acid and nitric acid, the peak shapes qualitatively resemble their respective current versus time functions. The positions of the dissolution peaks match the respective photocurrent versus time functions. On the other hand, in the case of methanesulfonic acid, maximum dissolution rates were measured after reaching the highest photocurrent density. These observations regarding the magnitude of the dissolution rates and dissolution peak shapes and positions indicate that different kinetics toward electrolyte decomposition has, in fact, a high impact on the stability of photoelectrodes. The formation of stable complexes seems to favor higher electrode degradation than OER. The low dissolution rate in nitric acid leads us to speculate that either OER or a radical with fast decay might be the reaction product in this electrolyte.

**Figure 4:**
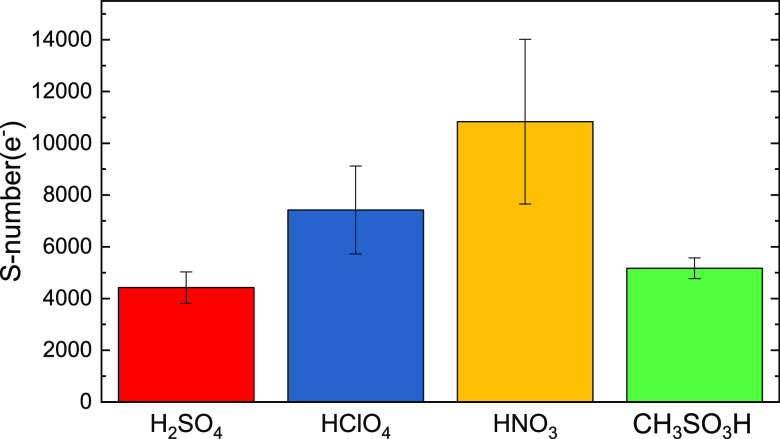
S-number(*e*^–^) = (*n*(*e*^–^)/*n*(W)) of WO_3_ thin films. The error bars are from five independent measurements.

However, currents in all electrolytes deviate strongly, and thus, the comparison of absolute dissolution might be misleading. In previous publications, the S-number concept was used to compare (photo)electrocatalyst stability.^26,28^ This concept, taken from OER electrocatalysis, estimates a faradaic efficiency of almost 100% toward OER at the anode side. The amount of generated oxygen is then divided by the amount of dissolved catalyst.^47^. However, as discussed before, the primary reaction on the photoanodes studied is not exclusively OER. Comparison based on the 100% faradaic efficiency of OER would be inconsistent. Therefore, we propose generalizing the S-number concept to any oxidated species at the anode side (Ox) and the catalyst’s metallic contents (M).
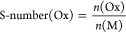
In this case, the reaction product on the anode side is not clearly defined. Therefore, it is useful to base S-number calculations on the number of transferred electrons *e*^–^. We define the S-number(*e*^–^) as
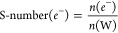
The S-number(*e*^–^) is related to the formerly used definition of the S-number(O_2_) by a factor of 4 and displayed for WO_3_ in all electrolytes in Figure 4.
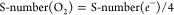


The S-numbers confirm the trend seen in the dissolution data. Relative to the charge transferred by photocurrent, WO_3_ electrodes are significantly more stable if operated with HClO_4_ and HNO_3_ than with H_2_SO_4_ or CH_4_O_3_S electrolyte. We speculate that the higher stability of WO_3_ electrodes in more stable electrolytes can be due to the fast detachment of intermediates (ClO_4_· and possibly NO_3_). Sulfuric acid and methanesulfonic acid, electrolytes that form stable persulfate complexes, might, on the other hand, complex dissolved W cations, enhancing dissolution.^48^ A similar effect of electrolytes on photoanode dissolution was recently shown for BiVO_4_.^28^ Such an effect is well-known in classical electrocatalysis, for example, for Pt that becomes significantly less stable in the presence of Cl^–^ anions.^49^ This higher dissolution is due to the formation of stable complexes preventing the redeposition of Pt, leading to higher measured dissolution rates. The complex formation has also been considered to be a significant phenomenon affecting the stability of photoelectrodes.^5,24,46^

In addition to complex formation, reactive intermediates (as products of the oxidation of anions in the electrolyte) adsorbed on the photoelectrode surface can also greatly influence its stability by changing the decomposition potential of WO_3_ (thus altering surface kinetics; see a more detailed explanation below).^24^ Compared to a former study of WO_3_ dissolution of electrodes formed from nanoparticles in sulfuric acid, the S-numbers visibly increase by a factor of 5 from 200 (S-number(O_2_),^26^) to 4000 (S-number(*e*^–^), this work). As mentioned above, structural differences can influence kinetics.^24^ The spray-coated photoelectrodes used in this study are more compact than samples prepared from nanoparticles. This suggests a high impact of sample preparation methods and electrode morphology on electrodes’ photoelectrochemical stability.

Finally, we want to venture a bit more into the reasons for the degradation of semiconductors in photoelectrochemical water splitting. The decomposition of a semiconductor occurs through a transition state (when the energy of holes reaches the surface back-bond energy of the semiconductor) that requires given activation energy. It is essential to note here that decomposition potentials and activation energies are always calculated for a structurally perfect bulk material. However, in reality, many factors can influence the activation energy and the decomposition potential, for example, defects on the electrode surface. The coordination of such sites is lower compared to the bulk making them thermodynamically prone to photocorrosion. Photocorrosion of the given semiconductor usually starts on these sites and accelerates as the concentration of defects increases. In addition to defects, the nature of the electrode/electrolyte interface (i.e., adsorbing/desorbing anions/cations, reaction intermediates, complexation, redox processes induced by photogenerated charge carriers, etc.) also plays a key role in photocorrosion.^24,25^ Based on theoretical calculations, WO_3_ should be thermodynamically susceptible to photocorrosion but kinetically stable (if no ions are involved in the process).^24^ However, as we have shown, kinetic barriers might be lowered in the presence of electrolyte anions, leading to the corrosion of the WO_3_ photoelectrode.

**Scheme 2:**
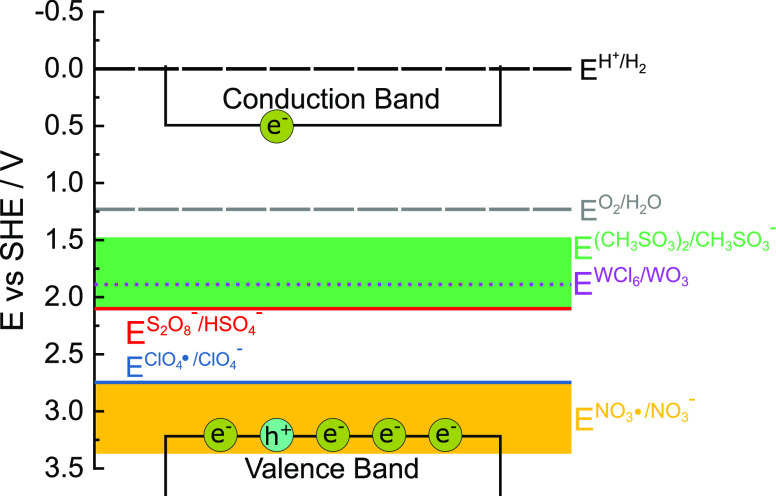
Schematic Display of Different Energy Levels of Electrolytic Redox Pairs with Respect to the Valence and Conduction Band of WO_3_ Narrow lines (red, blue) are taken from the literature. Broad (green, yellow) lines are estimated from stability data. The violet, dashed line indicates the decomposition of WO_3_ as estimated from theoretical calculations.^5^

Scheme 2 depicts the decomposition potentials of electrolytes used in this study in comparison to the thermodynamical potentials of water splitting and the band positions of WO_3_. The decomposition potentials of complex-forming electrolytes (H_2_SO_4_, CH_3_SO_3_H) are in a similar range as the decomposition potential of WO_3_ in the presence of Cl^–^ anions.^5^ Thus, it is likely that similar decomposition occurs in these electrolytes. On the other hand, the (estimated) potentials of HClO_4_ and HNO_3_ lie close to the valence band. Hence, the oxidation of the photoanode with these electrolytes is less likely.

## Conclusions

The stability of WO_3_ photoanodes toward photocorrosion was studied in different electrolytes. To prepare WO_3_ thin films, a new synthesis method based on a peroxotungstic acid route, but more suitable for upscaling, was suggested. The films, used as anodes in photoelectrochemical water splitting, displayed activities comparable to those shown previously in the literature. The maximum photocurrent densities were dependent on the electrolyte used, suggesting that OER is not the kinetically dominant charge transfer process on WO_3_. Based on previous reports, this difference in the maximum achievable photocurrents probably occurred due to the oxidation of the electrolyte anions.

While this is a serious issue from the perspective of a possible application, we additionally found that the presence of the different anions/their oxidation intermediates or products seriously affected the photostability of WO_3_, employing our PEC-ICP-MS setup. The stability of WO_3_ was decreased in the order of HNO_3_ > HClO_4_ > H_2_SO_4_ > CH_3_O_3_SH. The lowest stability was measured for electrolytes known to be oxidized by WO_3_ to form stable persulfate intermediates. We speculated that these intermediates either react with WO_3_ or form stable complexes, with W preventing its redeposition. Both scenarios lead to enhanced dissolution. Based on this study regarding stability and other literature reports on the Faradaic efficiency of WO_3_ toward PEC OER, HNO_3_ is the electrolyte ensuring high Faradaic efficiency toward OER in parallel with good photostability. Our results underline the fact that, in addition to thermodynamic considerations, kinetics also plays a key role in photoelectrode stability. This study marks the first step along the way. However, to fully understand the photocorrosion mechanism of WO_3_, further actions are needed involving the potential-dependent identification of the formed products/intermediates, mapping the formation and stability of W-based persulfate-containing complexes, etc. We strongly believe that our findings will stimulate a discussion within the community, which could eventually lead to new best practices (e.g., omitting the use of S-containing electrolyte anions), improved measurement protocols, and, in the end, better systems that fulfill the needs of potential industrial applications.

## Experimental Section

### Electrode Preparation

The peroxotungstic acid precursor was synthesized by dissolving 5 g of tungsten powder (fine powder 99+%, Merck) in 25 mL of H_2_O_2_ (30%, Merck).^19,23,30^ After the tungsten powder was completely dissolved, the beaker was filled with H_2_O (Merck, Milli-Q) and heated under continuous stirring to remove excess H_2_O_2_. When the remaining solution was reduced to 20 mL, heating was stopped, and the solution was diluted to 200 mL with isopropyl alcohol (IPA).

FTO glass slides (Sigma-Aldrich) were cleaned before spray coating by sequentially sonicating 10 min in 2% Hellmanex III solution, 10 min in DI water, and 10 min in IPA.

WO_3_ layers on FTO glass were prepared via spray coating the precursor using an ExactaCoat device (SonoTek) with an AccuMist spray nozzle (48 Hz). The hot plate temperature was adjusted to 80 °C, and the ink was sprayed with a flow rate of 0.33 mL min^–1^ at an ultrasonication power of 5 W, a nozzle height of 37 mm, and a traverse speed of 140 mm s^–1^ in a meander-shaped pattern with 1.5 mm pitch size. The electrodes were spray-coated 24 cycles.

After spray coating, the electrodes were calcinated at 500 °C for 1 h (2 K min^–1^ ramp).

### Characterization

#### XRD Measurements

The electrodes crystal structure was analyzed by X-ray diffraction (XRD) in Bragg–Brentano geometry using a Bruker D8 Advance equipped with a Cu Kα source and a LynxEye XE detector.

#### UV–Vis Measurements

Ultraviolet–visible absorption spectra were obtained using an optical spectrophotometer (OceanOptics) equipped with a deuterium–halogen light source (DH-2000-L) and an HR4000 spectrometer. The absorption spectra were obtained by subtracting the transmitted intensities from the incident intensity.

#### SEM Measurements

The surface analysis was performed via SEM imaging with a Zeiss Crossbeam 540 FIB-SEM microscope (focused ion beam scanning electron microscope) with a Gemini II column. Before imaging, the samples were attached to an aluminum SEM specimen stub with double-faced adhesive copper tape. Additionally, the samples were carbon-coated with a carbon sputter coater (Balzers Union, MED 010) for better conductivity. To further increase the conductivity of the region of interest, the WO_3_ surface was directly electrically connected to the FTO surface by the deposition of a small conductive platinum layer via ion beam deposition. The ion beam deposition was performed with a gas injection system (Orsay Physics, MonoGIS). Afterward, the SEM surface images were obtained with 3 kV acceleration voltage and a current of 750 pA.

The thickness of the WO_3_ layer was determined via FIB cross sections. Therefore, a protective platinum layer was deposited via ion beam deposition above the region of interest. Trenches were milled with an ion beam current of 3 nA and acceleration voltage of 30 kV. Finally, the SEM cross section images were obtained at 3 kV and 750 pA.

#### PEC-ICP-MS Measurements

Photoelectrochemical measurements were performed with a homemade photoelectrochemical scanning flow cell, made from polyether ethylene ketone. The cell was coupled to the counter electrode (graphite rod), the reference electrode (Metrohm Ag/AgCl, 3 M KCl), and an inductively coupled plasma mass spectrometer (ICP-MS; PerkinElmer NexION 300) by Tygon tubing.^26^ The working electrodes were placed on a freely movable, LabView-controlled XYZ stage (Physical Instruments), making contact with the cell opening (3.27 mm^2^ area). The working electrode was contacted by a wire. Electrochemical measurements were controlled by a Gamry 600 potentiostat. Electrolytes with a concentration of 0.1 M were mixed freshly every day from HClO_4_ (Merck Suprapur), H_2_SO_4_ (Merck Suprapur), HNO_3_ (Merck Suprapur), or CH_4_O_3_S (Acros Organics) and DI water (Merck, Milli-Q). The electrolyte reservoir was continuously purged with Ar (5.0, AirLiquide).

The electrodes were illuminated through an optical fiber patch cable (Thorlabs custom-made) with a 385 nm LED (Thorlabs M385F1) directly built into the cell. The power output of the LED was measured every measurement day (Thorlabs S120VC), calibrated with linear regression to LED input current, normalized to the illumination area of 4.5 mm^2^, and determined with UV-sensitive paper (Astromedia Solar-Fotopapier).

The ICP-MS was calibrated every day for W with freshly prepared standards (Merck Certipur) in the respective electrolyte with a four-point calibration (0; 0.5; 1; 5) μg L^–1^. Rhenium at a concentration of 10 μg L^–1^ served as an internal standard.
